# Developing Theoretically Based and Culturally Appropriate Interventions to Promote Hepatitis B Testing in 4 Asian American Populations, 2006–2011

**DOI:** 10.5888/pcd11.130245

**Published:** 2014-05-01

**Authors:** Annette E. Maxwell, Roshan Bastani, Beth A. Glenn, Victoria M. Taylor, Tung T. Nguyen, Susan L. Stewart, Nancy J. Burke, Moon S. Chen

**Affiliations:** Author Affiliations: Roshan Bastani, Beth A. Glenn, University of California, Los Angeles, California; Victoria M. Taylor, Fred Hutchinson Cancer Research Center, Seattle, Washington; Tung T. Nguyen, Nancy J. Burke, University of California, San Francisco, California; Susan L. Stewart, Moon S. Chen Jr., University of California, Davis, Sacramento, California.

## Abstract

**Introduction:**

Hepatitis B infection is 5 to 12 times more common among Asian Americans than in the general US population and is the leading cause of liver disease and liver cancer among Asians. The purpose of this article is to describe the step-by-step approach that we followed in community-based participatory research projects in 4 Asian American groups, conducted from 2006 through 2011 in California and Washington state to develop theoretically based and culturally appropriate interventions to promote hepatitis B testing. We provide examples to illustrate how intervention messages addressing identical theoretical constructs of the Health Behavior Framework were modified to be culturally appropriate for each community.

**Methods:**

Intervention approaches included mass media in the Vietnamese community, small-group educational sessions at churches in the Korean community, and home visits by lay health workers in the Hmong and Cambodian communities.

**Results:**

Use of the Health Behavior Framework allowed a systematic approach to intervention development across populations, resulting in 4 different culturally appropriate interventions that addressed the same set of theoretical constructs.

**Conclusions:**

The development of theory-based health promotion interventions for different populations will advance our understanding of which constructs are critical to modify specific health behaviors.

## Introduction

A theoretical foundation is crucial for understanding and predicting health behavior and for developing interventions to promote health. In addition, theory-based research allows for increased comparability of results across studies, populations, and health behaviors, and thus, for a more systematic approach to building the knowledge base. The consensus of the research community is that interventions should be culturally appropriate for the specific populations for which they are intended (1,2). Many culturally appropriate interventions to promote cancer screening have been developed during the last 2 decades (3–6). These interventions are usually developed in the language of the target population, depict members of the target population in print materials, and are delivered by staff or members from the target community. Members of the target group and community advisory boards are often asked to guide intervention development and to provide feedback on drafts of intervention programs. Research approaches that have been used for developing culturally appropriate interventions include community-based participatory research (5,7,8) and intervention mapping, a process that involves needs assessment, creating program objectives, selecting intervention methods and strategies, and designing a program (9–11). These and other articles have described the *process* of developing culturally specific interventions, usually for 1 ethnic group, but they provide little guidance for the development of culturally targeted, theory-based intervention *messages*.

A few community-based participatory research projects have focused on promoting hepatitis B testing among Asian Americans (12–16). Chronic hepatitis B infection is 5 to 12 times more common in Asian American populations than in the general US population and is the leading cause of liver disease and liver cancer among Asians (17,18). Hepatitis B testing is recommended for numerous high-risk populations, including Asian immigrants and their American-born children, because it can identify people infected who require treatment and people who have never been infected and require vaccination (19).

This article describes intervention components of 4 trials to promote hepatitis B testing that were conducted at 4 universities. All 4 trials received approval from the institutional review boards of the sponsoring universities: University of California Los Angeles for the Korean study, University of California San Francisco for the Vietnamese study, University of California Davis for the Hmong study, and Fred Hutchinson Cancer Research Center for the Cambodian study. Although 3 of the trials were funded through a National Cancer Institute program, 1 trial (Cambodian) was funded independently. All interventions were based on the Health Behavior Framework, which represents a synthesis of some of the major theoretical formulations in the area of health behavior (1,20). The purpose of this article was to describe the step-by-step approach that was followed in community-based participatory research projects in 4 Asian American communities to develop theoretically based and culturally appropriate interventions to promote hepatitis B testing. Specifically, we focus on the *content* of intervention materials that were developed for these 4 trials.

## Methods

First, we decided to implement the 4 interventions to promote hepatitis B testing in community settings rather than in clinical settings because a substantial proportion of each population did not proactively seek health care and had no regular source of care. For example, the proportion of study participants who had seen a doctor in the past 12 months ranged from 51% among Korean Americans to 63% among Cambodian Americans (21). For each population, we considered population characteristics such as age distribution, English proficiency, general level of education, immigration history, and affiliation with institutions such as faith-based centers. These characteristics can vary for different age groups and generations within each population. In addition, we reviewed intervention programs that had been implemented successfully in the population in the past. These factors guided the overall intervention approach in each group (eg, mass media, home visits by lay health workers).

Second, all 4 trials employed a common theoretical framework, the Health Behavior Framework, and study investigators collaborated closely during intervention development. The Health Behavior Framework is a comprehensive conceptual framework that posits that individual health behavior is influenced by a complex myriad of individual, health system, community, and society-level factors (Figure). We reviewed the literature to identify constructs of the Health Behavior Framework that had been associated with hepatitis B testing in prior studies and would therefore be important to address in our interventions (20). These studies primarily focused on modifying individual (eg, knowledge, health beliefs, patient-provider communication) and community level (eg, social norms) factors of the Health Behavior Framework. In an iterative process, we developed the intervention content to correspond to selected theoretical constructs of the Health Behavior Framework (1), incorporating community input to ensure that the interventions were culturally appropriate.

**Figure:**
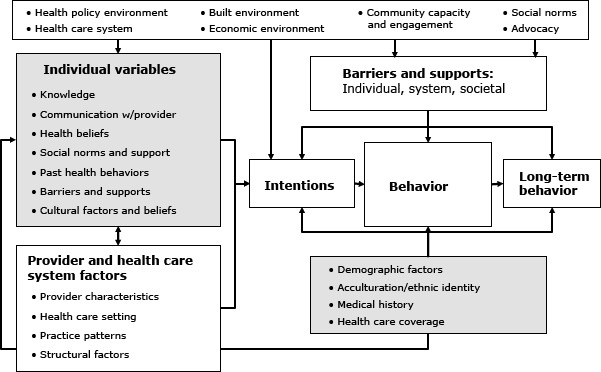
Health Behavior Framework. Reprinted with permission from Bastani R, Glenn BA, Taylor VM, Chen MS, Nguyen TT, Stewart SL, Maxwell AE. Integrating theory into community interventions to reduce liver cancer disparities: The Health Behavior Framework. Prev Med 2010, 50(1-2):63-67.

Third, we obtained community input for each component project through community advisory boards that met on a regular basis throughout the projects. The frequency of these meetings was dictated by the needs of the study and varied over time and by study. For example, we had monthly meetings in some of the studies during developmental phases and less frequent meetings (for example, twice a year) during data collection phases. Some studies had fewer meetings and more telephone calls to individual members of the advisory board to get input on a specific question. The Vietnamese advisory board included Vietnamese American physicians and nonphysician community members, and this same board had advised our study investigators in numerous prior studies. The Hmong study worked in close collaboration with the Hmong Women’s Heritage Association and a community advisory group of male leaders including a Hmong Western-trained physician in the Hmong community. The Korean advisory board included pastors, a pastor’s wife, elders, a church health program leader, and a Korean American physician. The Cambodian advisory group included Cambodian community leaders who worked for social and health services organizations serving Cambodian Americans.

In addition, each project had investigators or staff from the respective ethnic group, and intervention components were developed and pilot-tested with community members (15,22). Because each research team had unique prior experiences with their communities, the amount of pilot-testing conducted for each study varied widely as did the components that were pilot-tested and the number of community members that participated in pilot-testing. For example, the research team of the Vietnamese study had conducted prior mass-media interventions in the Vietnamese community; therefore, they focused on the pilot testing of new intervention components, such as a bilingual website for young Vietnamese Americans, by showing early versions of the website to members of their advisory committee and obtaining their feedback regarding the content, format, and graphics display.

All studies used a similar translation protocol consisting of development of a very simplified English version of the materials and forward translation into the respective language followed by back translation into English by a different person. Discrepancies between the original English version and the back translation were discussed by all bilingual team members, and community members were consulted if needed. If the English expression did not have an equivalent expression in one of the languages, the first course of action was to modify the English version. If that was not possible, we aimed for a translation that was not literal but conveyed the same concept. Together, these efforts ensured that all intervention messages and materials and study protocols were culturally sensitive and appropriate.

## Results

We developed intervention materials and messages for 4 community-based trials that included Vietnamese (N = 3,370), Hmong (N = 260), Cambodian (N = 250), and Korean Americans (N = 1,123). Sample sizes for each trial were based on study design and statistical power calculations, which in turn were based on the type of intervention and its anticipated magnitude of effect; all studies were designed to detect the specified effect size with 80% power at the 0.05 level, 2-sided.

The Vietnamese study used a quasi-experimental design to assess the effect of a media campaign on the prevalence of hepatitis B testing in an intervention community compared with a control community. We assumed an effect size of 10 percentage points (a 15 vs 5 percentage point increase in the intervention and control areas, respectively) on the basis of a previous study promoting hepatitis B vaccination (23), with an anticipated baseline prevalence of hepatitis B testing of 55% to 70%. Cross-sectional surveys were conducted in the intervention and control areas pre- and postintervention approximately 3 years apart. Households were selected by randomly sampling telephone numbers listed under Vietnamese surnames (12).

The Hmong and Cambodian studies used individually randomized designs to assess the effect of lay health worker interventions on receipt of a hepatitis B test among persons not previously tested. An effect size of 20 percentage points was expected in both studies, on the basis of the effect of a similar intervention on initial Papanicolaou testing (24). We also assumed that 5% to 20% of control group participants would report being tested and that 80% of participants would complete the study. Eligible participants were selected from households and randomized to intervention or control groups. In the Hmong study, households were randomly selected from a database created by the community collaborators; pre- and postintervention participant surveys were conducted 6 months apart (16). In the Cambodian study, households with eligible participants were identified by a previous community survey; a follow-up survey was conducted 6 months after randomization (13).

The Korean study used a group-randomized design to assess the effect of a small group educational intervention on receipt of a hepatitis B test among persons not previously tested. We assumed a clinically important effect size of 10 percentage points, an intraclass correlation of 0.05 (25), and 75% retention. The unit of randomization was Korean churches, with stratification by size and geographic location. Participants were recruited at the churches; pre- and postintervention participant surveys were conducted 6 months apart.

Given that all 4 populations comprised large proportions of immigrants who did not speak English well, all interventions were delivered in the relevant Asian language by native speakers from the target communities. Most print materials were bilingual so that predominantly English-speaking relatives or friends would also be able to understand the messages conveyed.

Population characteristics influenced the intervention approach that was chosen for each trial (Table 1). In addition, a review of the health promotion literature showed which intervention approach had been successfully used in each population before our trials. For example, mass media have been used in the Vietnamese community in tobacco control and cancer screening trials (26), and small group educational sessions have been conducted in Korean churches to promote breast cancer screening (27). In the 4 studies overall, interventions were delivered by using mass media (Vietnamese), lay health workers (Hmong and Cambodian), and small discussion group format (Korean), and, supplemented with small media (eg, radio spots) in all populations.

**Table 1:** Influence of Population Characteristics on Intervention Delivery Formats in Trials Promoting Hepatitis B Testing in 4 Asian American Populations

Population Characteristics	Previous Intervention Approaches	Selected Intervention Delivery Format
**Vietnamese American**		
Median age of the population is 34 years; 83% of adults are foreign-born; 50% speak English less than very well; 30% have less than a high school diploma, 21% have a high school diploma, 48% have more than a high school education (30). Most (98%) live in large communities in major urban centers that are served by Vietnamese-language media including print and radio and television stations.	Mass media campaigns were previously used in this community to reduce the rate of cigarette smoking among men and to increase breast and cervical cancer screening (4).	Media education campaign over 3 years: 30-second Vietnamese language paid television advertisements; 30 to 60-second Vietnamese language radio advertisements; bilingual Hepatitis B Internet website; newspaper articles and paid newspaper advertisements in Vietnamese language newspapers and in English language college campus newspapers; distribution of bilingual calendars, hepatitis B booklets, and hepatitis B info-cards at health fairs and community events.
**Hmong American**		
Median age of the population is 20 years; 67% of adults are foreign-born; 39% speak English less than very well; 36% have less than a high school diploma, 21% have a high school degree, 43% have more than a high school education; 21% of all families live below the Federal poverty level (30). Population originated from the mountainous regions of Vietnam, Cambodia, and Laos; Hmong are the most recent Asian American ethnic group to immigrate to the United States; at the time of migration, the Hmong were pre-literate, had religious beliefs based in animism, and were accustomed to a primarily agrarian lifestyle.	Lay-health-worker strategy has been used successfully in other Southeast Asian populations (26,29)	Home visits by lay health workers, use of flip chart and print materials in English and Hmong: Trained lay health workers from the Hmong community (1 man and 1 woman working together) visited Hmong households and led a discussion on hepatitis B using a bilingual flipchart and trifold. If desired, they scheduled an appointment for hepatitis B testing for participants and accompanied them to the testing site. Educational visits lasted, on average, 45 minutes
**Korean American**		
Median age of the population is 32 years; 78% of adults are foreign-born; 39% speak English less than very well; 23% are uninsured; 8% have less than a high school diploma, 18% have a high school diploma, 74% have more than a high school education (30). From 67% to 80% of Korean Americans regularly attend a Christian church (31). The church represents an important social institution within the Korean community. Many Korean Americans prefer to receive health information in a church setting.	Small-group educational sessions were conducted in churches to promote breast cancer screening among Korean American women (27)	Educational group session at churches and print materials: Trained Korean American lay people conducted a single-session, interactive, small-group educational discussion at Korean churches consisting of a multimedia presentation. Take-home print materials included a bilingual booklet, a resource guide listing hepatitis B testing facilities in the Los Angeles area, and an information brochure for physicians.
**Cambodian American**		
Median age of the population is 26 years; 77% of adults are foreign-born; 40% speak English less than very well; 20% are uninsured; 36% have less than a high school diploma, 24% have a high school diploma, 43% have more than a high school education; 20% of all families live below the Federal poverty level (30). Over 99% of Cambodian Americans immigrated to the United States over the last 3 decades or are the children of those immigrants. Low levels of acculturation to US norms of preventive medicine and limited English language proficiency preclude many Cambodian immigrants from receiving and understanding publicly disseminated information.	Lay-health-worker strategy had been used successfully in other Southeast Asian populations including Cambodian Americans (26,29)	Home visits by lay health workers and use of flip chart, educational pamphlet, and motivational DVD: Trained bilingual and bicultural Cambodian lay health workers conducted home visits that lasted an average of 45 minutes. Lay health workers and participants were matched by sex. During home visits, lay health workers used an educational flipchart to facilitate a discussion of hepatitis B, provided an educational booklet and a motivational DVD, and offered tailored counseling to address individual barriers to hepatitis B testing.

The 4 trials addressed Health Behavior Framework constructs (Table 2) (Table 3). Some constructs were addressed in very similar fashion in all 4 Asian American groups, and others had to be modified to be culturally appropriate, informed by community input. All 4 interventions provided information on the hepatitis B virus, hepatitis B transmission routes, and the hepatitis B test (Table 2). Many of the knowledge content areas were addressed with similar messages across the 4 populations (eg, knowledge of transmission routes). In some areas, content was customized on the basis of community input and pilot testing. For example, given the low educational level of the Hmong population and results from pilot testing, Hmong participants received more simplified messages than other groups and some basic information on the function of the liver. Although all studies explained that many people with hepatitis B infections had no symptoms, and therefore needed to be tested, the Hmong and the Cambodian studies also described potential symptoms to help explain hepatitis B infection and to distinguish it from other diseases. This was especially critical in these 2 populations, because some people confused hepatitis B and tuberculosis. The Vietnamese and Korean studies did not explain symptoms because pilot testing suggested that these 2 populations were more aware of hepatitis B and because we wanted to stress that everybody should get tested, even in the absence of symptoms. The hepatitis B test was not explained in messages developed for the Hmong population, but a photo was used to illustrate blood being drawn from the arm. Focus group findings informed the explanations and descriptions of the test that were provided to the Vietnamese and Korean populations. The Vietnamese explanation included the amount of blood that is needed for testing because focus groups revealed that some community members were concerned about losing too much blood. The Korean message explained that a hepatitis B test is not automatically included in routine blood testing (a misunderstanding that was revealed during pilot testing).

**Table 2:** Health Behavior Framework Constructs and Sample Messages Addressing Knowledge in 4 Asian American Populations

Health Behavior Framework Construct	Vietnamese American (Content of Bilingual Booklet)	Hmong American (Content of Flipchart)	Korean American (Content of Bilingual Booklet)	Cambodian American (Content of Flipchart)
**Knowledge of nature of hepatitis B^a^**	Hepatitis B is a contagious liver disease caused by hepatitis B. It can cause short-term and/or long-term liver inflammation (hepatitis), liver failure, cirrhosis and cancer. If you do have chronic hepatitis B infection, you should avoid alcohol and pain medications containing acetaminophen.	Hepatitis B can cause severe infection of the liver, liver cancer, and death. It is very small and you can’t see it with your naked eyes. *Photo of magnified virus*	Hepatitis B lives in the blood and other bodily fluids (saliva, pus, semen). Hepatitis B is a serious disease that spreads from person to person. Hepatitis B is 100 times more infectious than the AIDS virus. Hepatitis B can cause liver disease and lead to cancer if left untreated.	Hepatitis B is a swelling of the liver caused by a viral infection. The hepatitis B virus lives in the blood and other body fluids. People who are infected with the hepatitis B virus can pass it on to others. Hepatitis B can spread very easily.
**Knowledge of transmission routes of hepatitis B^b^**	You can get hepatitis B by coming into contact with an infected person’s bodily fluids (blood, saliva, pus, semen). Some ways you can get infected: Infected mother to baby during childbirth Having sex with an infected person without a condom Exposure to infected blood Using contaminated needles Sharing infected toothbrushes Sharing infected razors			
**Knowledge of symptoms of hepatitis B infection^c^**	Many people with hepatitis B do not know they have it because they feel healthy and do not yet have symptoms.	Feel tired, feel sick to your stomach, have a fever, do not want to eat, have stomach pain, have diarrhea; some people have dark-yellow urine, light-colored stools, and yellowish eyes and skin; many people do not have any symptoms and may feel fine. *Illustration of normal and jaundiced skin tone*	Most people don’t have any symptoms.	Most people who are infected with hepatitis B have no symptoms. Some people who are infected with hepatitis B have symptoms such as tiredness, loss of appetite, fever, nausea and vomiting, abdominal discomfort, and yellowish skin and eyes.
**Knowledge of function of liver^d^**	*Illustration of the abdominal cavity organs, including liver*	Helps digest food, absorb nutrients, fight infections and remove waste products and poisons from the body. *Illustration of the abdominal cavity organs, including liver*	No explanation, pictures, or illustrations provided	*Illustration of the abdominal cavity organs, including liver*
**Knowledge of hepatitis B test^e^**	The only way to know [if you are infected] is to get a hepatitis B blood test. About 1 teaspoon of blood is needed for the test. *Focus group finding: Concern in the Vietnamese community about the amount of blood that would be drawn for the test.*	There is a test available. *Photo: blood drawn from arm for hepatitis B test.*	It is a simple blood test. It is usually not included in routine blood testing, you need to ask your doctor specifically for a hepatitis B test. *Focus group finding: Misconception in the Korean community that Hepatitis B test is automatically done during routine blood testing.*	The only way for people to find out if they have been exposed to the hepatitis B virus is to have a blood test.

a Content similar for all groups; most simplified for Hmong Americans.

b Content similar for all groups – only example for Korean study is shown.

c Only 2 of the 4 studies described symptoms.

d Only Hmong study described basic liver function.

e Slightly different descriptions of hepatitis B test based on focus group findings.

**Table 3:** Health Behavior Framework Constructs and Sample Messages Addressing Communication With Provider and Health Beliefs In 4 Asian American Populations

Construct	Vietnamese American (Content of Bilingual Booklet)	Hmong American (Content of Flip Chart)	Korean American (Content of Bilingual Booklet)	Cambodian American (Content of Flipchart)
**Communication with provider:** 3 of the 4 populations were encouraged to show print materials to their doctor to facilitate communication. Hmong were assisted by lay health workers.	*Photo of patient and co-ethnic doctor discussing liver*. Bring this pamphlet to your doctor or clinic and ask for hepatitis B blood tests to check for hepatitis B surface antigen (HBsAg) and hepatitis B surface antibody (HBsAb). If you have never been infected with hepatitis B, you should ask the doctor to vaccinate you against it. The hepatitis B vaccine is very effective.	How do I request the test from my doctor? Kashia (Name of project in the community) can help you call a doctor to schedule an appointment for the blood test.	Please read the content of this booklet and make an appointment to get tested for hepatitis B today. Tell your doctor that you want to get tested for hepatitis B because it is common in your community and that you’re worried about it. Give your doctor the Hepatitis B Information for Physicians Brochure that is included in the take-home packet.	Most doctors will order a hepatitis B test if you ask for one. Tell the doctor you recently received information about hepatitis B from a community health worker. Tell the doctor you heard about the hepatitis B test and vaccine. Show the doctor your hepatitis B pamphlet.
**Perceived susceptibility:** Increased risk for hepatitis B infection was conveyed in a similar way for all groups	About 1 in 7 Vietnamese Americans has hepatitis B virus infection.	Anybody can get hepatitis B. Two of 10 Hmong know someone who is infected with hepatitis B. Hmong experienced 5 times higher incident rate of liver cancer than non-Hispanic whites.	*Graphs showing that 1 in 100 Americans is infected with hepatitis B, but 12 in 100 Koreans.* HBV is 100 times more infectious than the AIDS virus.	*Figures showing that Cambodians are much more likely to be infected with hepatitis B than other groups.* Hepatitis B infection is very common among Cambodians. Hepatitis B affects Cambodians over 10 times more than other groups of people.
**Perceived severity:** Similar content in all 4 groups but Vietnamese and Korean received more information than Hmong and Cambodian.	Can cause short-term and/or long-term liver inflammation (hepatitis), liver failure, cirrhosis and cancer. For chronic carriers: Without proper care, 1 in 4 people with chronic hepatitis B will eventually develop a fatal liver disease.	Hepatitis B can cause severe infection of the liver. Hepatitis B can cause liver cancer and death.	Hepatitis B causes 80% of liver cancer cases among Korean Americans. If untreated, it could cause liver disease and lead to liver cancer. The disease can damage your liver even without your knowledge.	Hepatitis B can cause serious health problems such as liver cancer.
**Cultural factors**	*Focus on family and community through multiple pictures of Vietnamese couples, families and groups*	*Photos of Hmong people, traditional attire, depiction of traditional activities (cultural performance art);* primary use of photos, graphics and visual art – very little text; delivery of intervention orally by lay health workers.	*Photos of Koreans evoking importance of family, pride in Korean culture (calligraphy, dancing, traditional attire).* Collectivism and focus on family benefits: “Now I can take action to protect my health and my family’s health.”	*Photo of Buddhist Temple in Cambodia.* Collectivism/focus on family benefits: “Knowing your hepatitis B test result can protect your family and help future generations of Cambodians.”
**Barriers/supports**	Most health insurance covers hepatitis B testing and vaccination. If you don’t have health insurance or cannot afford testing or vaccination, please contact the following partner agencies in your area to find out what services are available (list of local agencies).	Language barrier: Kashia can interpret for you at the appointment. Fear of finding hepatitis B infection: Kashia can help people who test positive for hepatitis B to get treatment and follow-up.	Many organizations offer this test at health fairs or through special programs. See the resource guide included in the take-home package. Lack of time: Losing time is losing a little, but losing your health is losing everything.	The hepatitis B blood test can be done at any doctor’s office or clinic, does not require any preparation, takes just a couple of minutes, and only requires a small amount of blood.

The trials addressed communication with providers and health beliefs (Table 3). Vietnamese, Korean, and Cambodian participants were advised to ask their doctor for a hepatitis B blood test and to show him or her the print materials they had received. In the Hmong study, lay health workers offered to schedule an appointment for participants and to accompany them to the health care provider or clinic because community partners suggested that this level of assistance would be required for the Hmong population to obtain hepatitis B testing. Health beliefs such as perceived susceptibility, perceived severity of hepatitis B infection, and cultural factors were addressed in similar ways across studies (Table 3). All projects addressed barriers to hepatitis B testing. However, barriers that had emerged in pilot testing were slightly different for each population. For example, lack of health insurance was addressed in the Vietnamese and the Korean populations, lack of time was specifically addressed in the Korean and the Cambodian populations, and language barriers and fear of finding hepatitis B infection were emphasized in the Hmong population.

## Discussion

We have described the process of developing interventions to promote hepatitis B testing for multiple Asian American populations and how theory-based constructs were addressed within these populations. Examples illustrate the extent and type of modification necessary to make the intervention approaches and messages culturally appropriate. Overall, good participation and retention rates in these trials suggest that the interventions were acceptable to all populations (12,13,16,28). Results regarding the efficacy of the 4 trials are consistent with the notion that the intervention approaches used were acceptable to the populations and were culturally appropriate. Intervention group participants were significantly more likely to report hepatitis B testing than control group participants at postintervention in the Hmong study (24% vs 10%) (16), in the Cambodian study (22% vs 3%) (13), and in the Korean study (19% vs 6%) (28). In the Vietnamese study, there was no significant increase in self-reported hepatitis B testing in the intervention group compared with the control group, but exposure to media elements was associated with receiving testing, and there was a borderline significant effect for planning to get hepatitis B testing in the intervention group compared with the control group (T. T. Nguyen, 2013, unpublished manuscript).

Both the intervention approach and the intervention content required cultural considerations. The intervention approach was chosen on the basis of population characteristics and was based on a literature review of health promotion interventions that had been successfully implemented in these populations in prior studies. For example, Vietnamese-language mass media are well established and have been used successfully in prior health promotion campaigns (4). The same lay-health-worker approach was used in the Cambodian and Hmong studies because both have small social networks in enclaves and lay-health-worker approaches have been shown to work with Cambodians (29). Intervention content was modified on the basis of cultural considerations and educational level of each population and was based on focus group findings and other pilot work. Although we have provided many examples to illustrate the rationale for the modifications of the intervention messages that were conveyed to the specific samples, we do not always have a clear explanation why some messages resonate more than others in a specific population. Even when we were lacking an explanation, we used the results of pilot testing and the advice of community experts as a guide to finalize materials.

Throughout the process of intervention development, the Health Behavior Framework provided a useful structure for developing culturally appropriate messages for all 4 ethnic groups. Using this framework ensured that all 4 trials addressed the same constructs that are thought to influence hepatitis B testing. We have previously shown that the relationships among Health Behavior Framework measures are generally consistent across the 4 Asian American groups and in the direction predicted by our theoretical framework (21). In this article, we highlight the value of the Health Behavior Framework for intervention development because it allowed a systematic approach to intervention development across populations.

Examples in this article are limited to 4 Asian American groups and the intervention approaches that were chosen for the 4 trials we conducted. Most of the intervention messages addressed individual factors. However, other intervention approaches and messages more focused on system- and community-level factors may also be acceptable and hold promise for promoting hepatitis B testing or other health behaviors in these and other populations.

The study makes a contribution to the field in several ways. It is one of the first articles to specifically describe the development of theoretically based interventions aimed at increasing hepatitis B testing with the long-term aim of reducing liver disease in Asian populations. Furthermore, it details how intervention messages addressing identical theoretical constructs were customized to meet the needs of 4 unique Asian populations.

Our examples demonstrate the utility of the Health Behavior Framework for developing interventions that are culturally appropriate for multiple Asian American populations. We encourage others to use similar methods to develop theory-based health promotion interventions that are culturally appropriate. This will advance our understanding of how to address key constructs underlying health behavior in diverse populations. Developing theory-based interventions across different populations will advance our understanding of which constructs are critical to modifying specific health behaviors.
